# Urinary Proteomics to Support Diagnosis of Stroke

**DOI:** 10.1371/journal.pone.0035879

**Published:** 2012-05-16

**Authors:** Jesse Dawson, Matthew Walters, Christian Delles, Harald Mischak, William Mullen

**Affiliations:** 1 Institute of Cardiovascular and Medical Sciences, College of Medicine, Veterinary & Life Sciences, University of Glasgow, Glasgow, United Kingdom; 2 Mosaiques Diagnostics, Hannover, Germany; I2MC INSERM UMR U1048, France

## Abstract

Accurate diagnosis in suspected ischaemic stroke can be difficult. We explored the urinary proteome in patients with stroke (n = 69), compared to controls (n = 33), and developed a biomarker model for the diagnosis of stroke. We performed capillary electrophoresis online coupled to micro-time-of-flight mass spectrometry. Potentially disease-specific peptides were identified and a classifier based on these was generated using support vector machine-based software. Candidate biomarkers were sequenced by liquid chromatography-tandem mass spectrometry. We developed two biomarker-based classifiers, employing 14 biomarkers (nominal p-value <0.004) or 35 biomarkers (nominal p-value <0.01). When tested on a blinded test set of 47 independent samples, the classification factor was significantly different between groups; for the 35 biomarker model, median value of the classifier was 0.49 (−0.30 to 1.25) in cases compared to −1.04 (IQR −1.86 to −0.09) in controls, p<0.001. The 35 biomarker classifier gave sensitivity of 56%, specificity was 93% and the AUC on ROC analysis was 0.86. This study supports the potential for urinary proteomic biomarker models to assist with the diagnosis of acute stroke in those with mild symptoms. We now plan to refine further and explore the clinical utility of such a test in large prospective clinical trials.

## Introduction

Prompt and accurate diagnosis is crucial for the effective management of ischaemic stroke and transient ischaemic attack (TIA). Both are clinical diagnoses, supported by imaging findings. Even when patients are assessed by a specialist in cerebrovascular medicine, as many as one fifth of patients initially thought to have a stroke [1], and one half of patients initially felt to have had a TIA [2], eventually receive an alternate diagnosis.

Clinical assessment tools can facilitate accurate diagnosis and are increasingly used in the routine evaluation of patients with suspected stroke [2], [3], [4], yielding diagnostic accuracy in the range of 80–90%. The use of brain imaging, particularly magnetic resonance imaging (MRI), can provide further certainty and in the setting of stroke is required to distinguish ischaemia from haemorrhage. In one study, where MRI with diffusion weighted imaging (DW-MRI) was directly compared to CT [5], sensitivity of MRI for the detection of acute cerebral ischaemia was ≈ 83%. Although this is vastly superior to non-contrast CT in the detection of acute ischaemia (sensitivity 16%), the false negative rate of MRI approximates to 17%, and it cannot be performed in all patients [5].

The ability rapidly to confirm the presence of stroke, particular minor ischaemic stroke or TIA and in the significant number of patients in whom there is early diagnostic doubt would be advantageous [6], [7]. One potential approach is the use of proteomics, which involves the simultaneous analysis of thousands of proteins and peptides. Changes in the expression of several proteins have been described in brain extracellular fluid and plasma of those with acute stroke [8], [9]. Recently, urinary proteomic biomarker models have been developed and showed potential for accurate identification of those with, or at high risk of, cardiovascular disorders such as ischaemic heart disease [10], diabetes, diabetic nephropathy [11] and pre-eclampsia [12]. We hypothesised that a urinary proteomic biomarker model could be developed to reliably identify those with minor ischaemic stroke or TIA (those most likely to have inconclusive brain imaging) and that biomarkers would be discovered which were associated with stroke severity. We developed such a biomarker model in a cohort of patients with minor ischaemic stroke and a control population with excess cardiovascular risk but no history of recent stroke or TIA.

## Results

### Demographic Variables

Urine samples were available from 65 cases and 41 controls. All samples available were included in the proteomics study. There were differences between the case and control groups in baseline characteristics **(** **table** **1** **)**. Cases had a lower frequency of hypertension and use of alpha-blockers and calcium channel blockers. However, all except one control and nine cases were taking at least one anti-hypertensive drug. Of cases, 20 (31.3%) suffered TIA, the remainder had suffered stroke and 40 (61.5%) had findings compatible with cerebrovascular disease on brain imaging although acute cerebral infarction was only demonstrated in 10 (16.1%) cases. CT was the most commonly performed imaging modality. Partial anterior circulation stroke was the commonest subtype.

**Table 1:** Baseline Characteristics.

Variable	Cases	Controls	P Value
Age, years	70.1 (10.6)	66.5 (11.2)	0.100
Female Sex	31 (47.7%)	17 (40.5%)	0.552
Stroke	44 (68.8%)	–	–
TIA	20 (31.3%)	–	–
Total Anterior Circulation Stroke	6 (9.4%)	–	–
Partial Anterior Circulation Stroke	30 (46.9%)	–	–
Lacunar Stroke	18 (28.1%)	–	–
Posterior Circulation Stroke	1 (1.6%)	–	–
NIHSS Score (median, IQR)	2 (2–4)	–	–
Smoker	20 (30.8%)	6 (14.3%)	0.066
Ischaemic Heart Disease	20 (30.8%)	11 (26.2%)	0.667
Previous CVA	19 (29.2%)	0	<0.001
PVD	2 (3.1%)	3 (7.1%)	0.379
Diabetes Mellitus	4 (6.2%)	8 (19.0%)	0.058
Hyperlipidaemia	14 (21.5%)	8 (19.0%)	0.811
Hypertension	34 (52.3%)	36 (85.7%)	<0.001
Atrial Fibrillation	6 (9.2%)	2 (4.8%)	0.477
Dipyridamole Therapy	13.8 (9%)	1 (2.4%)	0.085
ACE I or ARB Therapy	36 (55.4%)	22 (52.4%)	1
Diuretic Therapy	24 (36.9%)	22 (52.4%)	0.161
Beta-blocker Therapy	23 (35.4%)	20 (47.6%)	0.231
CCB Therapy	23 (33.8%)	23 (54.8%)	0.045
Alpha-blocker Therapy	2 (3.1%)	8 (19.0%)	0.013
Any Antihypertensive Therapy	56 (86%)	41 (98%)	0.085
Statin Therapy	50 (76.9%)	31 (73.8%)	1

For continuous variables, values given are mean (SD) unless stated.

### Biomarkers for Presence of Stroke or TIA

All samples were analyzed with CE-MS, and processed to result in an individual list of peptides and proteins present in each sample, as well as normalized ion counts as measure for relative abundance. To identify potential biomarkers for stroke, 26 control samples and 33 cases were randomly selected. This left a second, blinded test set, consisting of the remaining 47 samples. These were analyzed under identical conditions. This approach has been proven superior in the past to avoid bias introduced due to analysis of training- and test-set under slightly different conditions.

**Figure 1:**
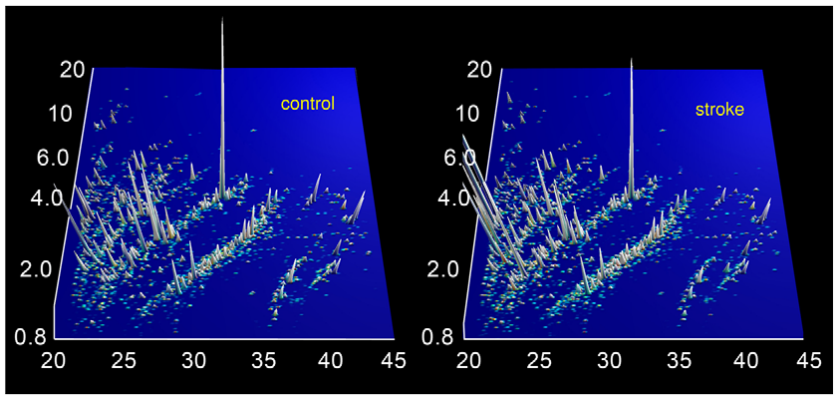
Urinary polypeptide signatures in cases and controls. Normalized molecular weight (800–20 000 Da) in logarithmic scale is plotted against normalized migration time (18–45 minutes). The mean signal intensity of the polypeptide peak is given in 3-dimensional depiction. n = 26 controls and 33 cases.

**Figure 2:**
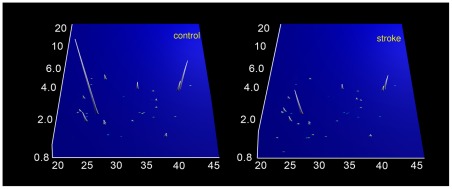
Polypeptide signatures of the 35 biomarker model in cases and controls. Normalized molecular weight (800–20 000 Da) in logarithmic scale is plotted against normalized migration time (18–45 minutes). The mean signal intensity of the polypeptide peak is given in 3-dimensional depiction. n = 26 controls and 33 cases.

The compiled proteomics data of the 26 control and 33 case samples was used to identify potential biomarkers and also to establish a classifier (the biomarker model). These data are shown in **figure** **1** **.** Only two biomarkers were identified that also passed rigorous adjustment for multiple testing **(** **table** **2** **).** Since multidimensional classifier models based on only 2 biomarkers perform poorly, we selected additional candidates. Two classifiers based on either 14 potential biomarkers (all peptides in the dataset with a nominal p-value <0.004) or 35 potential biomarkers (all peptides in the dataset with a nominal p-value <0.01) were established. The distribution of these 35 potential biomarkers is shown in **figure** **2** **.**

**Table 2:** Proteins included in biomarker models. Mass is measure in Daltons.

Mass	CE time	Protein Name	Protein Sequence	P value
**896.5**	**22.5**	**Gamma-glutamyltranspeptidase 2**	**VAVPGEIRG**	<0.001 *
1164.5	26.1	FXYD domain-containing ion transport regulator 4	DPFANKDDPF	0.005
1170.6	26.4	Beta-2-microglobulin	SDLSFSKDWS	0.007
1170.6	21.2	Hemoglobin subunit alpha	VLSPADKTNVK	0.009
1194.6	29.2	Collagen alpha-1(I) chain	GDRGEpGPpGPAG	<0.001 *
1210.4	36.5	Protein Phosphatase 1 regulatory subunit 3F isoform 1	GGGEGSTDGGmSPS	0.009
1240.6	27.1	Inter-alpha-trypsin inhibitor heavy chain H4	FSVMPGLKMTM	0.007
**1532.6**	**26.4**	**Collagen alpha-1(II) chain**	**RDGEPGTPGNpGPpGP**	0.003 *
1541.6	24.5	Fibrinogen alpha chain	DEAGSEADHEGTHST	<0.001 *
1576.6	26.4	FRAS1-related extracellular matrix protein 3	RPSFMATmMmEVD	0.005
1640.6	23.2	–	–	0.004
1640.7	31.0	Collagen alpha-3(IX) chain	AAGAGLDGpEGDQGpQGp	<0.001 *
1773.82	34.6	Isoform 1 of Collagen alpha-1(IV) chain	GppGPPGppGPPGEKGQMG	<0.001 *
1786.6	38.3	–	–	0.003 *
1794.7	23.3	–	–	<0.001
1911.1	25.0	Uromodulin	SGSVIDQSRVLNLGPITR	0.005 *
1912.9	32.7	Collagen alpha-1 (I) chain	NGApGNDGAKGDAGApGApGSQ	0.004 *
1925.8	23.2	Isoform 1 of Fibrinogen alpha chain	PGSPRPGSTGTWNPGSSER	0.003 *
1949.9	21.7	Collagen alpha-1 (I) chain	GDDGEAGKpGRpGERGPPGp	0.002
1952.9	32.2	Collagen alpha-2(I) chain	GEKGpSGEAGTAGPpGTpGPQG	0.005
1956.1	33.0	Ceruloplasmin (ferroxidase)	NGRQKDVDKEFYLFPT	0.005
2007.9	22.1	Collagen alpha-1 (III) chain	DGESGRpGRpGERGLpGPpG	0.005
2168.6	35.1	–	–	0.008
2548.3	35.2	AGT Angiotensinogen	FAVYDQSATALHFLGRVANPLSTA	0.009 *
2607.2	34.4	Collagen alpha-1(XXVII) chain	GSKGQpGDSGEMGFpGmAGLFGPKGPP	0.001
2682.1	22.5	–	–	0.009
2791.3	29.5	–	–	0.009
3157.1	34.7	–	–	0.002 *
3223.4	39.1	–	–	0.007
3292.5	39.4	–	–	<0.001 *
3359.6	31.9	Collagen alpha-1 (I) chain	PpGADGQPGAKGEpGDAGAKGDAGPpGPAGPAGPpGPIG	0.008
3583.6	41.5	–	–	0.002 *
3681.5	23.5	–	–	0.006
4218.0	26.1	Polymeric-immunoglobulin receptor	EEKAVADTRDQADGSRASVDSGSSEEQGGSSRALVSTLVPLG	0.006
4263.0	23.5	–	–	0.010

CE-time  =  capillary electrophoresis migration time. All proteins were included in the M35p001 model. *  =  included in the M14 sig model. The 2 biomarkers that passed adjustment for multiple testing are labeled in bold.

**Figure 3:**
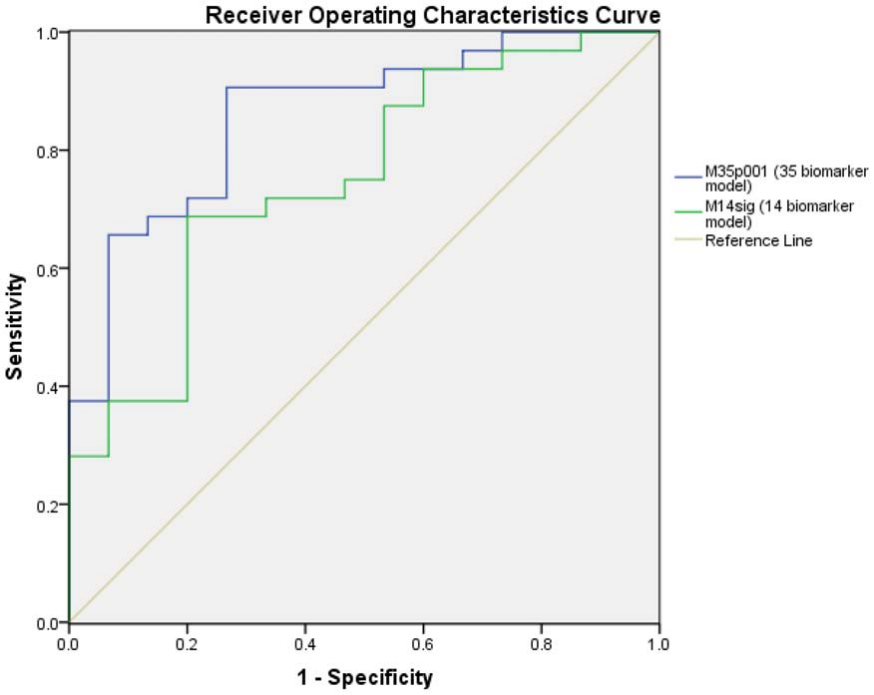
Receiver operating characteristics curve for the 14 and 35 biomarker models.

When tested on the blinded test set of 47 samples (32 cases, 15 controls), the proteomic signal was significantly different between groups; in the 14 biomarker model, median value of the classifier was 0.96 (−1.04 to 3.3) in cases compared to −1.06 (IQR −3.18 to 0.35) in controls, p = 0.006; in the 35 biomarker model, median value of the classifier was 0.49 (−0.30 to 1.25) in cases compared to −1.04 (IQR −1.86 to −0.09) in controls, p<0.001. The 14 biomarker model had sensitivity of 75% and specificity of 73% for identification of stroke and gave an area under the curve (AUC) of 0.75 on ROC analysis. The 35 biomarker model performed better; sensitivity was 56%, specificity was 93% and the AUC on ROC analysis was 0.86 **(** **figure 3** **)**.

### Peptide Sequences of Identified Biomarkers

We were able to sequence and identify several of the peptides included in the biomarker models **(on-line appendix)**. These included FXYD domain-containing ion transport regulator 4, inter-alpha-trypsin inhibitor heavy chain H4, uromodulin, polymeric-immunoglobulin receptor and collagen fragments.

### Biomarkers Associated with Severity of Stroke

**Figure 4:**
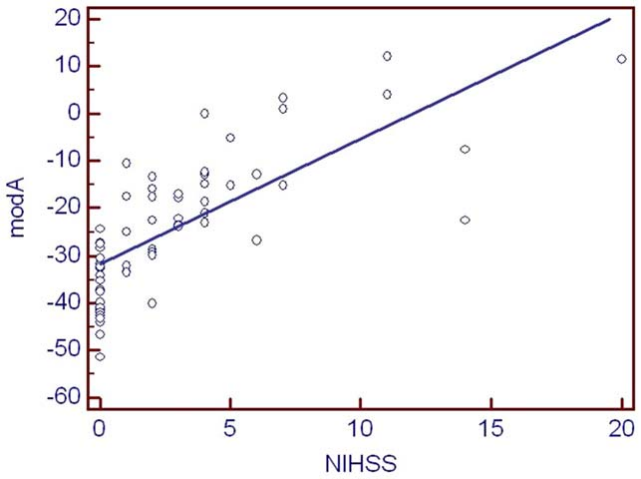
Scatter plot of National Institute of Health of Stroke Scale (NIHSS) score and the biomarker model classifier (modA).

To identify potential biomarkers associated with severity of stroke, all proteomic data from cases were included in the analysis. A total of 4453 urinary proteins and peptides detected in the samples were investigated for their correlation with the NIHSS score. Several urinary peptides correlated with the NIHSS score, although only at a moderately high level. Since only the data from patients with stroke were examined, the correlated peptides showed essentially no overlap with the potential diagnostic peptides that differentiate between stroke and control; only one peptide was identical in both sets. In an attempt to develop a classifier that may enable classification of the patients with respect to disease severity, several of the potential biomarkers were combined using linear combination of the normalized amplitude of the single biomarkers. Such linear combination generally does not result in “overfitting”, as it is one-dimensional only. When subsequently testing the classifier based on the 34 potential biomarkers that showed the highest correlation, it was strongly correlated with NIHSS score (r = 0.74, 95% CI 0.60 to 0.84, p = <0.0001) **(** **figure 4** **)**.

## Discussion

We explored the urinary proteome in patients with acute stroke or TIA and in a group of controls with elevated cardiovascular risk. We were able to identify a panel of potential urinary peptide biomarkers, for two of these we could establish significant differences in a small cohort of patients with acute stroke or TIA in comparison to a control group. When combining several of these potential biomarkers to an SVM-based classifier / biomarker model, this classifier was associated with stroke with very high significance, and enabled separating of a second blinded cohort with good accuracy; an AUC of 0.86. These results indicate that urinary biomarkers enable the detection of stroke in the population at risk. As already demonstrated previously [10], [13], the data also clearly indicate that classifiers based on a larger number of biomarkers enable higher accurate classification, are significantly superior to single or only few biomarker-based classifiers. This is in part owed to biological variability; while the urinary peptides can generally be analyzed with high accuracy, their level is to a substantial degree subject to biological variability, which can be counteracted by combining an array of biomarker peptides [14]. Further, we were able to identify a number of peptides which correlated with stroke severity. Importantly, this was achieved in a cohort of patients with minor stroke and TIA. When combing these biomarker to a linear classifier, very good correlation with the NIHSS score could be obtained. Thus, as well as assisting with diagnosis, our data suggest proteomic changes related to severity of stroke which if confirmed could provide mechanistic insights and help with risk stratification after ischaemic stroke.

The diagnosis of stroke and TIA can be difficult. The use of clinical scoring systems and brain imaging can enhance sensitivity and diagnostic accuracy. CT imaging performs poorly in this setting with reported sensitivity of 16% early after ischaemic stroke [5] and we found similar in our study. Crucially, even with MRI imaging, sensitivity does not reach 100%; even with use of diffusion weighted MRI imaging meaning novel means to facilitate diagnosis would be a tremendous advantage. Key predictors of false-negative diffusion weighted imaging include a brainstem location of stroke and lower NIHSS score [6]. In a further study, 25% of patients with mild stroke or stroke-like deficits had a negative DWI study [7]. Thus, while MRI is the imaging modality of choice, it has limitations; sensitivity is not 100%, particularly after mild stroke or TIA and around 10% of patients are unable to undergo MRI because of contraindications including electronic implants, medical instability, agitation and claustrophobia. Further, MRI is not always available and in many countries non-contrast CT remains the initial imaging modality of choice. In our study, mean NIHSS score was 3.1 suggesting our biomarker model may enhance diagnostic certainty in exactly the group where even diffusion weighted MRI is not definitive. This is the first study to show that models based on the urinary proteomic signal have potential to assist with the diagnosis of stroke. Although sensitivity of our model was 54%, specificity was high and further studies will aim to improve model performance.

We have developed a biomarker model using urine samples. This would carry several advantages over a plasma based model. First, the urinary proteome is more stable that than in plasma. This raises the possibility of community based sampling, in cases of suspected mild stroke in rural environments which is less feasible for plasma proteome analysis. We found the biomarker model with 35 included biomarkers performed best, which is in line with previous observations where classifiers based on a larger number of biomarkers result in increased accuracy [10].

We were able to sequence a number of the peptides in the biomarker models, several of which may have roles in the pathophysiology of acute ischaemic stroke. For example, these included one which has recently been shown by other groups to be under-expressed in acute ischaemic stroke (inter-alpha-trypsin inhibitor heavy chain H4) [15]. FXYD-4 (CHIF) is a regulator of Na-K-ATPase [16]. Dysfunction of such ion pumps is a key feature of the cytotoxic oedema which occurs in acute ischaemic stroke and expression of FXYD-4 is altered in renal ischaemic injury [17]. Polymeric-immunoglobulin receptor is known to be expressed in neuronal tissue and may be implicated in protein transcytosis across the blood brain barrier [18]. Blood brain barrier dysfunction is also a pathological feature of acute ischaemic stroke. Phosphatases have been implicated in the pathogenesis of other brain disorders [19] and gamma-glutamyl transpeptidase is present on the apical surface of endothelial cells at the blood brain barrier and expression increases in animal models of stroke [20]. An angiotensinogen peptide also differed between controls and cases. There are several possible explanations for this. The renin – angiotensin system is involved both in the pathogenesis of hypertension, is modulated by numerous anti-hypertensive agents but is also a critical mediator of ischaemic injury in acute stroke [21]. Given the use of most anti-hypertensive drugs was similar and hypertension was common in both groups, this difference may well reflect the ischaemic injury in the case group. Uromodulin levels were slightly lower in cases. There are recent reports that uromodulin may have a role in blood pressure regulation and uric acid metabolism [22], both of which link with outcome after stroke. Further, uromodulin may induce inflammatory cytokine production [23]. Several collagen fragments were predictive of stroke. Whilst the exact biological function of collagen fragments is unclear in acute stroke, it is unsurprising that collagen be affected given its abundance in extracellular matrix of blood vessel adventitia. Given that some collagen fragments were increased in cases and others were reduced, it is possible that these changes reflect levels of an as yet unidentified protease. Our biomarker models are therefore based on peptides with clear biologic plausibility and provide potential mechanistic insights worthy of further evaluation.

There are weaknesses to consider. This study is not a comparison of the urinary proteome between those presenting with suspected stroke who have stroke mimic with those who have confirmed stroke. However, rather than a healthy control group, our control group was recruited from cardiovascular risk factor clinics and most had hypertension and a substantial number had established ischaemic heart disease. The ability of the biomarker model to distinguish those with stroke from non-stroke patients at high cardiovascular risk suggests that it may perform well when tested in those with stroke mimic. Participants were originally recruited to a study of aspirin resistance which makes generalization of our results an important consideration. However, since inclusion and exclusion criteria were wide, with the exception of the requirement for pre-existing aspirin use, we believe our results are generalizable.

There were some differences between the case and control groups. Cases had a higher incidence of previous stroke, and lower incidence of hypertension and lower usage of calcium channel blockers. It is possible, but unlikely, that these differences will have confounded results. A substantial minority of our cases had suffered TIA; albeit a TIA deemed severe enough to warrant admission to hospital. The proteomic signal in those with NIHSS of 0 was similar to that in controls suggesting that our model may perform best in those with minor stroke (where it would still be clinically useful) rather than in those with suspected TIA. We found a correlation between 34 biomarkers and NIHSS score but this was a mild cohort of stroke patients and this finding needs to be replicated in a wider population of stroke patients.

Our study was small, but it enabled identification of biomarkers and the generation of classifiers that also were validated in independent blinded samples, following recommended procedures for proteomic analysis [24]. While the data show a clear and highly significant association of the biomarkers and models with stroke, it is evident that the results require further refinement and evaluation in larger prospective studies where we can fully assess the potential role in clinical practice.

In summary, we have developed a urinary proteomic biomarker model which shows potential to assist with the diagnosis of acute stroke in those with mild symptoms, and in the assessment of severity. We now plan to explore the clinical utility of such a test in large prospective clinical trials.

## Materials and Methods

### Study Population

The study was performed in the Institute of Cardiovascular and Medical Sciences at the University of Glasgow. The study protocol was approved by the Scottish Multicenter Research Ethics Committee A. All participants gave written informed consent before participation in the study. Samples were obtained during a study of the prevalence of aspirin resistance in those with acute stroke [25]. In this study, all participants gave blood samples for platelet function analysis and a proportion gave their first urine sample following admission.

Cases were aged >18 years, were taking aspirin therapy, and had a diagnosis of ischaemic stroke or transient ischaemic attack. All participants were recruited within 24 hours of onset of symptoms and the first urine passed thereafter was used. Urine samples were thus all obtained within 24 hours (median time from recruitment to obtaining urine sample 20 minutes, IQR 0 to 60 minutes) and before further drug treatment was used. Those with brain imaging which suggested intracerebral haemorrhage were excluded. Cases were identified on admission to the Acute Stroke Unit and gave informed consent. Where potential participants were unable to consent, assent from the nearest relative or welfare guardian was accepted. Consecutive patients meeting these criteria were included. We followed our well established procedures for identifying those who have suffered stroke or TIA. All participants referred to our unit are reviewed within 24 hours of first contact by a Consultant Stroke Physician and standard investigations organized. Clinical features, imaging findings and diagnosis is then reviewed in a multi-disciplinary team meeting which includes 6 Consultant Stroke Physicians, a Radiologist and a Neurologist. It is at this meeting that a final consensus diagnosis of stroke or non-stroke is applied.

Controls comprised individuals aged over 18 years of age who were taking aspirin therapy. They were required to be at excess cardiovascular risk, defined as attending a secondary care specialist cardiovascular risk factor clinic. They were required to have taken aspirin for a minimum of one year, to never to have suffered a cardiovascular event on aspirin and to never have suffered stroke or stroke like symptoms. Exclusion criteria were the same as for cases (with the exception of the need for brain imaging). All gave informed consent and were mostly identified during out-patient attendance at local out-patient clinics.

All urine samples were immediately frozen at −70°C.

### Sample preparation

Sample preparation was performed as described previously [26]. In short, a 0.7 mL aliquot was thawed immediately before use and diluted with 0.7 mL 2 M urea, 10 mM NH4OH containing 0.02% SDS. To remove proteins of higher molecular mass the sample was filtered using a Centrisart ultracentrifugation filter device (20 kDa MW cut-off; Sartorius, Goettingen, Germany) at 3,000 rcf until 1.1 ml filtrate was obtained. The filtrate was then loaded onto a PD-10 desalting column (GE Healthcare, Sweden) and equilibrated in 0.01% NH4OH in HPLC-grade H2O (Roth, Germany) to decrease matrix effects by removing urea, electrolytes, and salts, and also to enrich polypeptides. Finally, samples were lyophilized and stored at 4°C. Shortly before CE-MS analysis, lyophilisates were resuspended in HPLC-grade H20 to a final protein concentration of 0.8 µg/µL verified by the BCA test (Interchim, Montlucon, France).

### Capillary Electrophoresis-Mass Spectometry (CE-MS) analysis

All samples available were analysed with CE-MS in accordance with recently published guidelines for clinical proteome analysis [24]. CE-MS analysis was performed as previously described using a P/ACE MDQ capillary electrophoresis system (Beckman Coulter, Fullerton, USA) on-line coupled to a MicroTOF MS (Bruker Daltonic, Bremen, Germany) [12]. The ESI sprayer (Agilent Technologies, Palo Alto, USA) was grounded, and the ion spray interface potential was set between −4.0 and −4.5 kV. Data acquisition and MS acquisition methods were automatically controlled by the CE via contact-close-relays. Spectra were accumulated every 3 s over a range of m/z 350 to 3000. Details on accuracy, precision, selectivity, sensitivity, reproducibility, and stability of the CE-MS method have been provided previously [26], [27].

### Data processing

Mass spectral ion peaks representing identical molecules at different charge states were deconvoluted into single masses using MosaiquesVisu software [28]. Only those signals with z >1 that were observed in a minimum of 2 consecutive spectra with signal-to-noise ratios >4 were included. The software employs a probabilistic clustering algorithm and uses both isotopic distribution as well as conjugated masses for charge-state determination of peptides/proteins. The resulting peak list characterizes each polypeptide by its molecular mass, CE-migration time, and ion signal intensity (amplitude) value. We applied a calibration method for CE-migration time and ion signal intensity on the basis of “internal standard” peptides, which we proved to be superior over creatinine normalization [29], [30]. In addition, this method of standardization is not affected by renal function, hence no adjustments for reduced GFR are required [30] All detected peptides were deposited, matched, and annotated in a Microsoft SQL database, allowing further analysis and comparison of multiple samples [31]. Peptides were considered identical within different samples, when mass deviation was lower than 50 ppm for small peptides or 75 ppm for larger peptides and proteins. Due to analyte diffusion, CE peak widths increase with CE migration time. In the data clustering process this effect was compensated by linearly increasing cluster widths over the entire electropherogram (19 to 45 min) from 2 to 5%. After calibration, deviation of migration time was controlled to be below 0.45 min.

### Establishment of stroke-specific classifiers

In order to develop the biomarker models that differed between cases and controls, a random selection of 33 cases and 26 controls was used. The remaining samples were then used to internally validate the biomarker models. Biomarker models were generated using the support-vector-machine (SVM) based MosaCluster software [10], [12]. The software generates multimarker models by combination of polypeptides that are differentially distributed between case and control samples. In SVM, a urine sample is regarded as a p-dimensional vector, p being the number of peptides included in the pattern. The SVM algorithm constructs a (p−1) –dimensional separation plane between case and control vectors. From all possible hyperplanes that separate cases and controls, the one with the largest distance to the nearest data points on both sides is selected. Sample classification itself is performed by determining the Euclidian distance of a particular data set to the maximal margin of the hyperplane and assignment of a positive or negative value depending on which side of the hyperplane, case or control, the vector is located.

To develop a biomarker model related to stroke severity, the normalized logarithmic amplitudes of significantly correlated markers (nominal p-value <0.01, r>0.33) were combined additive in a linear model, as described [27].

### Sequencing of peptides

The urine samples were analysed on a Dionex Ultimate 3000 RSLS nano flow system (Dionex, Camberly UK). The samples (5 μl) were loaded onto a Dionex 100 μm×2 cm 5 μm C18 nano trap column at a flowrate of 5 μl/min by a Ultimate 3000 RS autosampler (Dionex, Camberley UK) The composition of the loading solution was 0.1% formic acid and acetonitrile (98∶2). Once loaded onto the trap column the sample was then washed off into an Acclaim PepMap C18 nano column 75 μm×15 cm, 2 μm 100 Å at a flowrate of 0.3 μm/min. The trap and nano flow column were maintained at 35°C in a column oven in the Ultimate 3000 RSLC. The samples were eluted with a gradient of solvent A: 0.1% formic acid verses solvent B: acetonitrile starting at 5% B rising to 50% B over 100 min. The column was washed using 90% B before being equilibrated prior to the next sample being loaded. The eluant from the column was directed to a Proxeon nano spray ESI source (Thermo Fisher Hemel UK) operating in positive ion mode then into an Orbitrap Velos FTMS. The ionisation voltage was 2.5 kV and the capillary temperature was 200°C. The mass spectrometer was operated in MS/MS mode scanning from 380 to 2000 amu. The top 10 multiply charged ions were selected from each full scan for MS/MS analysis, the fragmentation method was HCD at 35% collision energy. The ions were selected for MS2 using a data dependant method with a repeat count of 1 and repeat and exclusion time of 15 s. Precursor ions with a charge state of 1 were rejected. The resolution of ions in MS1 was 60,000 and 7,500 for HCD MS2.

Data files from experiments performed on the HCD-enabled LTQ were searched against the IPI rat non-redundant database using the Open Mass Spectrometry Search Algorithm (OMSSA, http://pubchem.ncbi.nlm.nih.gov/omssa) and SEQUEST (by using Thermo Proteome Discoverer), without any enzyme specificity. No fixed modification was selected, and oxidation of methionine and proline were set as variable modifications. Mass error window of 10 ppm and 0.05 Da were allowed for MS and MS/MS, respectively. In the case of SEQUEST, the peptide data were extracted using high peptide confidence and top one peptide rank filters.

For further validation of obtained peptide identifications, the strict correlation between peptide charge at pH of 2 and CE-migration time was utilized to minimize false-positive identification rates [32]: Calculated CE-migration time of the sequence candidate based on its peptide sequence was compared to the experimental migration time.

### Statistical analysis

Descriptive statistics were used to describe the data. After testing for normal distribution, continuous data were compared by Wilcoxon t-test, as this test has proven to be of superior statistical power in proteomics datasets [33]. A *P*-value <0.05 was considered to be statistically significant. *P*-values were calculated using the natural-logarithm transformed intensities and the Gaussian approximation to the t-distribution. In order to control the false discovery rate, the scores were ranked and significance thresholds were adjusted by the Benjamini and Hochberg method [13]. Once candidate biomarker models were identified, their sensitivity, specificity and area under the receiver operating characteristic (ROC) curve were calculated. The relationship between potential biomarkers and stroke severity measured by the National Institute of Health Stroke Scale was compared using the spearman correlation co-efficient.
